# Supplementary figures and images for: Identification of m6A-associated autophagy genes in non-alcoholic fatty liver
Source: PeerJ. 2024 Feb 29;12:e17011. doi: 10.7717/peerj.17011 (PMC10909346; doi:10.7717/peerj.17011)

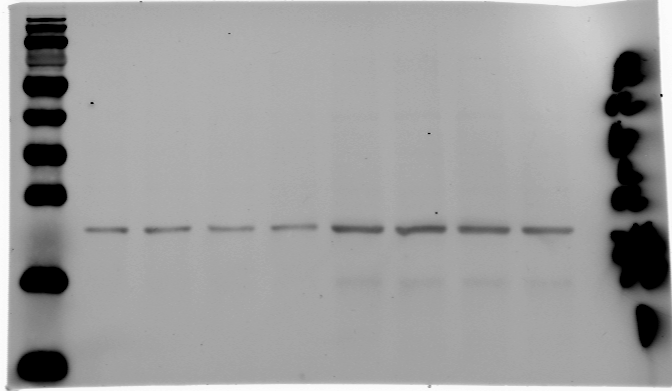

Supplement: Supplemental Information 1 [file peerj-12-17011-s001.tif]

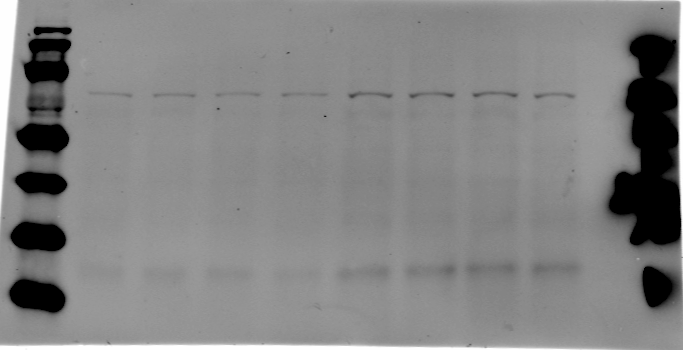

Supplement: Supplemental Information 2 [file peerj-12-17011-s002.tif]

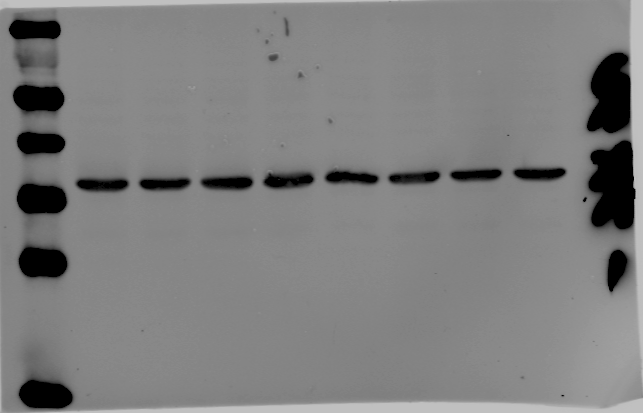

Supplement: Supplemental Information 3 [file peerj-12-17011-s003.tif]

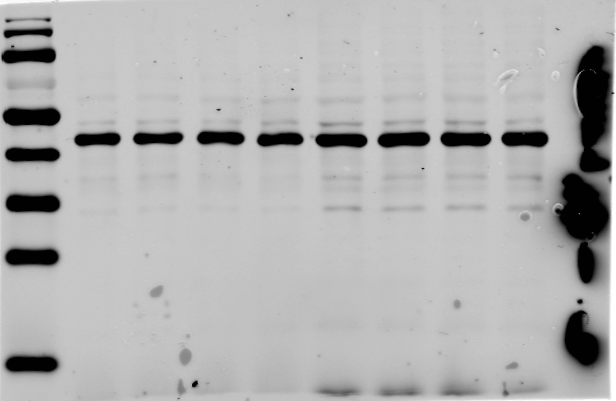

Supplement: Supplemental Information 5 [file peerj-12-17011-s005.tif]
